# Universals and variations in moral decisions made in 42 countries by 70,000 participants

**DOI:** 10.1073/pnas.1911517117

**Published:** 2020-01-21

**Authors:** Edmond Awad, Sohan Dsouza, Azim Shariff, Iyad Rahwan, Jean-François Bonnefon

**Affiliations:** ^a^Department of Economics, University of Exeter Business School, Exeter EX4 4PU, United Kingdom;; ^b^The Media Lab, Massachusetts Institute of Technology, Cambridge, MA 02139;; ^c^Department of Psychology, University of British Columbia, Vancouver, BC V6T1Z4, Canada;; ^d^Centre for Humans & Machines, Max-Planck Institute for Human Development, Berlin 14195, Germany;; ^e^Institute for Data, Systems, and Society, Massachusetts Institute of Technology, Cambridge, MA 02139;; ^f^Toulouse School of Economics, Toulouse School of Management-Research, Centre National de la Recherche Scientifique, University of Toulouse Capitole, Toulouse 31015, France

**Keywords:** morality, dilemma, culture

## Abstract

When do people find it acceptable to sacrifice one life to save many? Cross-cultural studies suggested a complex pattern of universals and variations in the way people approach this question, but data were often based on small samples from a small number of countries outside of the Western world. Here we analyze responses to three sacrificial dilemmas by 70,000 participants in 10 languages and 42 countries. In every country, the three dilemmas displayed the same qualitative ordering of sacrifice acceptability, suggesting that this ordering is best explained by basic cognitive processes rather than cultural norms. The quantitative acceptability of each sacrifice, however, showed substantial country-level variations. We show that low relational mobility (where people are more cautious about not alienating their current social partners) is strongly associated with the rejection of sacrifices for the greater good (especially for Eastern countries), which may be explained by the signaling value of this rejection. We make our dataset fully available as a public resource for researchers studying universals and variations in human morality.

## Significance

We report the largest cross-cultural study of moral preferences in sacrificial dilemmas, that is, the circumstances under which people find it acceptable to sacrifice one life to save several. On the basis of 70,000 responses to three dilemmas, collected in 10 languages and 42 countries, we document a universal qualitative pattern of preferences together with substantial country-level variations in the strength of these preferences. In particular, we document a strong association between low relational mobility (where people are more cautious about not alienating their current social partners) and the tendency to reject sacrifices for the greater good—which may be explained by the positive social signal sent by such a rejection. We make our dataset publicly available for researchers.

Every culture has rules about what is right or wrong, but they often disagree on the particulars of moral decisions. Moral universals are difficult to find, as they often reveal some degree of cultural variation upon closer inspection. For example, most people think that accidental transgressions are not as bad as intentional transgressions, but the importance of this distinction varies across cultures, to the point of disappearing in some small-scale societies (1, 2). Likewise, most people refrain from acting in a purely self-interested manner in economic games, but different cultures have different expectations about what constitutes fair behavior in these games (3). Likewise again, every culture prohibits at least some form of homicide, while disagreeing about which exact form of homicide is wrong (4, 5): Different cultures can have different views on what counts as self-defense or provocation, or on the offenses that should be punished with capital execution.

Accordingly, studying moral judgment at a global scale is important for theoretical development. When researchers attempt to develop a theory of moral psychology based on findings that replicate in a handful of countries, they can find it challenging to determine whether the findings reflect basic, universal cognitive processes or some similarities in social and cultural contexts (6). The larger and the more diverse the set of countries in which the findings hold, however, the greater the appeal of theories based on basic cognitive processes or universal moral grammars (7–9). Conversely, when researchers attempt to develop theory on the basis of findings that differ in a handful of countries, they can find it challenging to pinpoint the exact cultural features that may explain these differences, since any two countries can differ on many cultural traits. The larger and more diverse the set of countries, the easier the task becomes.

**Fig. 1.**
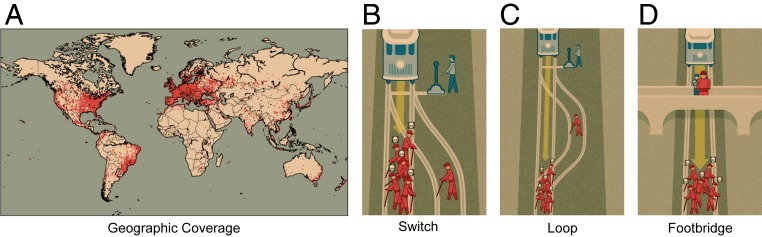
Geographical coverage and stimuli. (*A*) Many participants came from Europe and the Americas, but more than 40 countries delivered sample sizes of 200+ participants. Participants made decisions in three scenario variants: (*B*) Switch, (*C*) Loop, and (*D*) Footbridge.

In this article, we report on the moral universals and variations in responses to three variants of the trolley problem (10, 11), one of the focal points of contemporary moral psychology (12). Based on the responses of 70,000 participants, collected in 10 languages and 42 countries with a lower bound of 200 responses per scenario and country (Fig. 1*A*), we firmly consolidate some results of previous comparative surveys, and provide evidence of cultural correlates that were not possible to assess from other, smaller datasets. To this end, we leveraged the popularity of the “Moral Machine” website (moralmachine.mit.edu). While the Moral Machine website was primarily designed to explore the ethics of self-driving cars (13), it also offered a “Classic” mode, where visitors made decisions about the traditional *Switch*, *Loop,* and *Footbridge* variants of the trolley problem; these are the data that we report in this article.

In the first scenario illustrated in Fig. 1*B* (the Switch), a trolley is about to kill five workers, but can be redirected to a different track, in which case it will kill one worker. In the third scenario illustrated in Fig. 1*D* (the Footbridge), a large man can be pushed in front of the trolley. The large man will die, but his body will stop the trolley before it can kill the five workers on the track. There are two important differences between Switch and Footbridge. First, the death of the worker in Switch is not instrumental in saving the five—it is an incidental yet foreseeable side effect of the action that saves the five. In contrast, in Footbridge*,* the death of the large man is instrumental in saving the five—his death would not be a side effect, but a means to save them (10, 11). Second, the sacrifice of the large man in Footbridge requires the use of personal force against him, whereas no personal force is exerted against anyone in Switch (14, 15). These two factors make it psychologically more acceptable to act in Switch than in Footbridge. In the Loop scenario illustrated in Fig. 1*C*, the trolley can be redirected to a different track, where it will kill one worker whose body will stop the trolley before it can kill the five. Personal force is not required against anyone, and many find it difficult to decide whether the death of the worker is intended or merely foreseen (16, 17). As a result, people tend to place the acceptability of acting in Loop somewhere in between that of Switch and Footbridge, a pattern of moral preferences we will simply call Switch–Loop–Footbridge.

The first objective of this article is to consolidate the universality of the qualitative Switch–Loop–Footbridge pattern. Indeed, despite the complexity of the Switch–Loop–Footbridge preferences, and that of their emotional and cognitive correlates (15, 18–20), there is evidence that their ranking might be universal. The preference for Switch over Footbridge is already well documented. Because this preference is large, it is easy to detect even with small samples, and it was, indeed, detected in at least 30 countries (21–25). The difference between Switch and Loop is smaller and requires larger sample sizes. For example, one survey using large random samples of 1,000 participants established this difference in the United States, China, and Russia (24). The “Many Labs 2” project (25) obtained preferences for Switch, Loop, and Footbridge in 19 countries, the most diverse dataset so far. Switch was significantly preferred to Loop in 11 countries (with a median sample size n=149 per scenario). Switch was nonsignificantly preferred to Loop in six countries (median n=59 per scenario), and two countries (median n=39 per scenario) showed a nonsignificant difference in the opposite direction. The difference between Loop and Footbridge is typically larger, and was detected as significant in 16 out of 19 countries.

Fewer data are available regarding the second objective of this article, which is to document cultural variations in the quantitative acceptance of sacrifice in Switch*,* Loop*,* and Footbridge. Even if the relative ranking of these preferences turns out to be constant, different countries may endorse sacrifice at different absolute levels. Some previous comparative studies tested whether the difference between Switch and Footbridge was moderated by culture, and did not find evidence for such a moderation (21, 22, 25). Other studies, however, directly compared the rates at which participants in different countries endorsed sacrifice. Russian participants in one such study endorsed sacrifice less in all three scenarios, compared to a mixed sample of American, Canadian, and British participants (23), but this difference was not replicated in a larger sample comparing Russian and American participants (24). This latter study, however, found that Chinese participants endorsed sacrifice less in all three scenarios, compared to Russian or American participants—and another study found that Chinese participants were less likely than British participants to endorse sacrifice in Switch (26).

In this article, we report data from a much broader range of countries, allowing us to explore the cultural correlates of the willingness to sacrifice in Switch*,* Loop*,* and Footbridge. In *Results*, we will document the correlations between moral preferences and a standard set of country-level variables (individualism, religiosity, and gross domestic product)—but we want to emphasize the theoretical interest of one specific variable, *relational mobility*. Relational mobility refers to the fluidity with which people can develop new relationships (27, 28). In societies with low relational mobility, people develop lifelong relationships but have few options to develop new ones. As a result, they show greater social cautiousness, in order to avoid conflict in existing relationships (29). In contrast, in societies with high relational mobility, people have many options to find new social partners, which makes it easier to leave old friends behind and replace them with new friends (30). This flexibility may explain why people in high relational mobility societies show greater self-disclosure, which, in turn, makes it easier to end up with like-minded friends (31).

Accordingly, holding attitudes that put one at social risk is especially costly in low relational mobility societies, where alienating one’s current social partners is harder to recover from. This cost is likely lower (although not absent) in high relational mobility societies, as they offer abundant options to find new, like-minded partners. Accordingly, people in low relational mobility societies may be less likely to express and even hold attitudes that send a negative social signal. Endorsing sacrifice in the trolley problem is just such an attitude. Recent research has shown that people who endorse sacrifice in the trolley problem are perceived as less trustworthy, and less likely to be chosen as social partners (32–34). As a consequence, low relational mobility societies may feature more acute pressure against holding this unpopular opinion. Although it is possible that this pressure would discourage people who hold socially risky positions from expressing them, it could also change people’s attitudes, making certain ideas morally “unthinkable.”

This conjecture is not trivial, for at least two reasons. First, it assumes that the preference for sacrifice in the trolley problem sends a negative social signal in low relational mobility societies, but the effect has only been shown so far in Western, high relational mobility societies. Second, it assumes that people in high relational mobility societies, such as those of the North Atlantic, have less to lose by holding unpopular opinions—linking relational mobility to other frequently discussed East–West differences (35). Alternatively, in an environment with a more fluid interpersonal marketplace, people would compete more to be chosen as relational partners, and would thus experience greater pressure against holding opinions that mark them as untrustworthy. Our large and diverse dataset provides the ideal testing grounds for clarifying the association between relational mobility and moral decisions.

Indeed, we collected the largest and most diverse dataset so far on moral decisions about the three main variants of the trolley problem, recording responses from 70,000 participants in 10 languages and more than 40 countries (36). This dataset allows us to provide unprecedented insights about cultural universals and differences in moral psychology. First, it reveals a universal pattern of support for the Switch–Loop–Footbridge spectrum that is suggestive of a common underlying cognitive structure. Second, the systematic variability between groups and its relation to other cross-national variables of interest speaks to recent debates about the ecological validity of the trolley problem (37, 38).

## Results

**Fig. 2.**
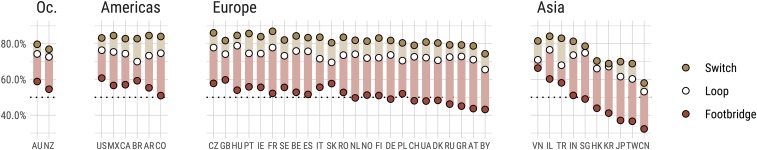
Percentage choosing to sacrifice in each scenario variant. In all of these countries, participants were most likely to sacrifice in Switch, then in Loop, then in Footbridge. Within each continent, countries are ordered by decreasing order of the average acceptability of sacrifice in the three scenarios. Oc., Oceania. AU: Australia, NZ: New Zealand, US: United States of America, MX: Mexico, CA: Canada, BR: Brazil, AR: Argentina, CO: Colombia, CZ: Czechia, GB: United Kingdom of Great Britain and Northern Ireland, HU: Hungary, PT: Portugal, IE: Ireland, FR: France, SE: Sweden, BE: Belgium, ES: Spain, IT: Italy, SK: Slovakia, RO: Romania, NL: Netherlands, NO: Norway, FI: Finland, DE: Germany, PL: Poland, CH: Switzerland, UA: Ukraine, DK: Denmark, RU: Russian Federation, GR: Greece, AT: Austria, BY: Belarus, VN: Viet Nam, IL: Israel, TR: Turkey, IN: India, SG: Singapore, HK: Hong Kong, KR: The Republic of Korea, JP: Japan, TW: Taiwan (Province of China), CN: China.

Results provide the best available evidence so far for the universality of the Switch–Loop–Footbridge pattern. As shown in Fig. 2, every country in our dataset showed the same pattern of responses: Participants endorsed sacrifice more for Switch (country-level average: 81%) than for Loop (country-level average: 72%), and for Loop more than for Footbridge (country-level average: 51%). These figures are consistent with recent data (39) showing that the moral acceptability of sacrifice in Footbridge-type dilemmas has steadily increased in the last few decades, reaching about 45 to 60% for participants born after 1990—but we note that this high acceptability could also be due to other demographic characteristics of our sample (see *Materials and Methods* and *SI Appendix* for further details).

**Fig. 3.**
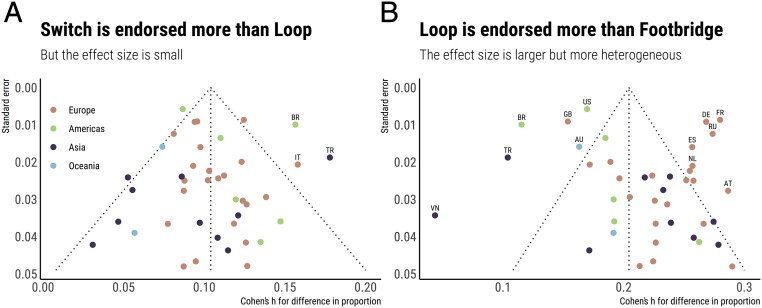
Funnel plots for the difference in decisions between scenario variants. (*A*) The difference between the proportion of participants choosing to sacrifice in Switch and the proportion choosing to sacrifice in Loop was small, with an effect size converging to about 0.10 as a function of increasing sample size. (*B*) The difference between the proportion of participants choosing to sacrifice in Loop and the proportion choosing to sacrifice in Footbridge was twice as large, but also heterogeneous, with greater variance in countries with greater sample size.

As shown in Fig. 3*A*, the difference between the endorsement of sacrifice in Switch and in Loop was small, with an effect size of about 0.10 (Cohen’s h). While there were substantial national differences in this effect for countries with a small sample size, these differences gradually disappeared for countries with greater sample sizes (and, thus, greater statistical power). This convergence suggests that the psychological difference between Switch and Loop might be relatively homogeneous across the world. In contrast, Fig. 3*B* shows a different pattern for the difference between Loop and Footbridge. Here we can see that heterogeneity between countries increases for countries with greater sample sizes. This suggests that responses to the Footbridge problem are subject to strong cultural influences.

Previous research (reviewed in the Introduction) suggested that participants from Eastern countries would be less likely to endorse sacrifice in trolley problems than participants from Western countries. These surveys often compared China or Japan to the United States. Here, we reproduce these findings, with an important nuance. As shown in Fig. 2, it is true, in our data, that participants from the United States are much more likely to endorse sacrifice than participants in China or Japan—however, it is also true that the United States is among the Western countries most likely to endorse sacrifice, while China and Japan are among the Eastern countries least likely to endorse sacrifice. As a result, surveys that focused on a US–China or US–Japan comparison might have overestimated the magnitude of the Western–Eastern difference. Asian countries, in particular, show large variations in the baseline acceptability of sacrifice in all scenario variants—but this is also true, to a lesser extent, of Western countries.

**Fig. 4.**
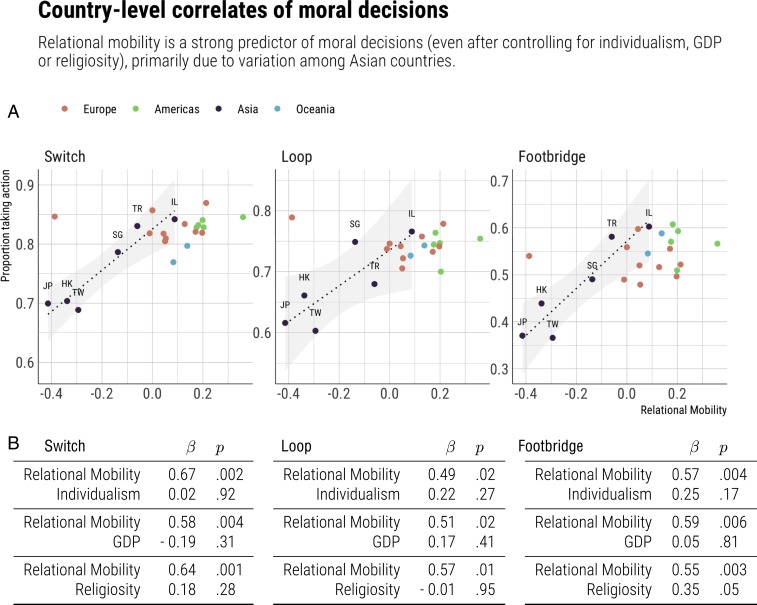
Association between relational mobility and decisions in the three scenario variants. (*A*) Scatter plots and (*B*) tables with regression model coefficients and *P* values show that greater relational mobility is correlated with a greater propensity to endorse sacrifice in all variants, even after controlling for other relevant country-level variables. This correlation is especially strong among the subsample of Asian countries, as shown by the regression lines which were fitted to this subsample for illustration.

Our large dataset allowed us to further explore these cultural variations. We explore a wider array of these in *SI Appendix*, but, as explained in the Introduction, we focused on the association between relational mobility and the propensity to endorse sacrifice in each scenario variant. As shown in Fig. 4*A*, this association is positive. Relational mobility was positively correlated with the propensity to endorse sacrifice in Switch ($r_{21}=0.67$, p<0.001, 95% CI 0.36 to 0.85), in Loop ($r_{21}=0.56$, p=0.005, 95% CI 0.20 to 0.79), and in Footbridge ($r_{21}=0.64$, p<0.001, 95% CI 0.32 to 0.83). These associations were robust in regression models (Fig. 4*B*) that controlled for individualism, gross domestic product, and religiosity.* Interestingly, the effect of relational mobility was mostly driven by Asian countries, which span the lower half of relational mobility. In Asian countries, the correlation between relational mobility and taking action was 0.95 for Switch (p=0.004, 95% CI 0.59 to 0.99), 0.83 for Loop (p=0.04, 95% CI 0.05 to 0.98), and 0.93 for Footbridge (p=0.007, 95% CI 0.50 to 0.99). Non-Asian countries, meanwhile, cluster high in both relational mobility and propensity to sacrifice, as would be expected on the basis of the trend revealed by the Asian countries.

## Discussion

Most people would agree that killing is wrong—but perhaps not in all circumstances. Homicide is such a grave act that it is of considerable psychological interest to understand the contexts in which people may tolerate the killing of another human. For at least two decades now, moral psychologists have been collecting data on one such context: When do people find it acceptable to sacrifice one life to save many? These data suggested a complex pattern of universals and variations in the way different cultures tackle this question. On the one hand, data suggested that people from different cultures displayed a remarkable qualitative regularity, the Switch–Loop–Footbridge ordered pattern of preferences. On the other hand, data also suggested that people from different cultures displayed quantitative variations in the exact degree to which they endorsed sacrifice in each of these scenarios—mostly such that people from Eastern countries showed less acceptance than people from Western countries. One limitation of these findings is that they were often based on small samples from a small number of countries outside of the Western world. Thanks to the popularity of the Moral Machine website, we were able to collect data at a much greater scale.

Our sample is large but by no means ideal. We relied on voluntary participation in a viral online experiment, and our sample shows clear signs of self-selection. As described in *Materials and Methods* and detailed in *SI Appendix*, our sample is skewed in terms of age, gender, and education: We estimate that a third of our participants were young, college-educated men. Thus, our population is more diverse than a convenience sample of university students, but does not optimally reflect the diversity of the countries we collected data from. We fully acknowledge this limitation, but we note that, to the best of our knowledge, no research group has ever attempted to collect data on moral preferences from nationally representative samples in 42 countries.

With this caveat, our data provide the best existing evidence that people universally endorse sacrifice in Switch more than in Loop, and in Loop more than in Footbridge. Given this universal result, it seems appropriate to explain the qualitative Switch–Loop–Footbridge pattern in terms of basic cognitive processes, rather than to seek explanations based on cultural norms. In contrast, we observed substantial country-level variations in the quantitative acceptability of each sacrifice. Our data replicated previous results, such as the lower acceptability observed in China compared to the United States—but they also allowed us to explore country-level variations at a finer granularity, for example, by going beyond East–West differences and focusing on variations between Eastern countries.

We focused our analysis on relational mobility, because of its theoretical interest, but one limitation of this strategy is that relational mobility has not been estimated yet in all of the countries represented in our dataset. However, since we are making our dataset public, researchers will be able to update our analyses once relational mobility is measured in new countries—or conduct new analyses based on any country-level variable which may be associated with cultural variations in moral preferences. Indeed, we believe that an important contribution of this article is the public resource we are making available to the scientific community: a dataset containing moral preferences expressed by 70,000 participants in 10 languages and more than 40 countries.

## Materials and Methods

Data collection started in June 2017, when we deployed a Classic mode as part of the Moral Machine website.^†^ This Classic mode only offered three scenarios (Switch, Loop, Footbridge). The three scenarios were graphically designed to retain a distinct Moral Machine visual identity while using a sepia color palette, which distinguished them from the futuristic Moral Machine scenarios featuring autonomous vehicles. While the Moral Machine (and its Classic mode) is still collecting data, this paper reports results obtained during the first 20 months of data collection, up until March 2019.

Users of the Classic mode are presented with the three standard trolley scenarios, in a random order. We restricted our sample to users who gave a response to all three scenarios, and to countries in which at least 200 users did so. Each scenario is introduced with the question “What should the man in blue do?” Two images shown side by side show the two possible decisions and their outcomes. They are augmented with a text description, as follows (the words Switch, Loop, and Footbridge are not shown to participants):

**Switch.** A man in blue is standing by the railroad tracks when he notices an empty boxcar rolling out of control. It is moving so fast that anyone it hits will die. Ahead on the main track are five people. There is one person standing on a side track that doesn’t rejoin the main track. If the man in blue does nothing, the boxcar will hit the five people on the main track, but not the one person on the side track. If the man in blue flips a switch next to him, it will divert the boxcar to the side track where it will hit the one person, and not hit the five people on the main track.
**Loop.** A man in blue is standing by the railroad tracks when he notices an empty boxcar rolling out of control. It is moving so fast that anyone it hits will die. Ahead on the main track are five people. There is one person standing on a side track that loops back toward the five people. If the man in blue does nothing, the boxcar will hit the five people on the main track, but not the one person on the side track. If the man in blue flips a switch next to him, it will divert the boxcar to the side track where it will hit the one person and grind to a halt, thereby not looping around and killing the five people on the main track.
**Footbridge.** A man in blue is standing on a footbridge over the railroad tracks when he notices an empty boxcar rolling out of control. It is moving so fast that anyone it hits will die. Ahead on the track are five people. There is a large person standing near the man in blue on the footbridge, and this large person weighs enough that the boxcar would slow down if it hit him (the man in blue does not weigh enough to slow down the boxcar). If the man in blue does nothing, the boxcar will hit the five people on the track. If the man in blue pushes the one person, that one person will fall onto the track, where the boxcar will hit the one person, slow down because of the one person, and not hit the five people farther down the track.

Using a translation and back-translation process, we made this description available in Arabic, Chinese, English, French, German, Japanese, Korean, Portuguese, Russian, and Spanish. The country from which users accessed the website was geolocalized through the IP address of their computer or mobile device. After completing the three-scenario session, users had an opportunity to share the link to the experiment with their social network, and were presented with an optional survey of their demographic, political, and religious characteristics. About 20,000 users opted to fill out that survey, and their responses gave us an approximate estimation of the composition of the sample. This composition deviated from the general population in several ways: 75% of survey takers were men, 75% were younger than 32, and 73% were college-educated (overall, 36% of survey takers were young, college-educated men). Their political beliefs were skewed toward the left (with a mean score of 37 on a 0 to 100 scale from progressive to conservative), and their religiosity was skewed toward secular (with a mean score of 25 on a 0 to 100 scale from nonreligious to religious). See *SI Appendix* for a complete description of the sample of survey takers as well as an analysis of the impact of these characteristics on their decisions.

### Data Availability.

All of the data and code used in this article have been deposited in the Open Science Framework, and can be accessed using the following website: https://bit.ly/2Y7Brr9.

## Supplementary Material

Supplementary File

## Footnotes

*See *SI Appendix* for zero-order correlations with and between these variables, as well as four additional country-level variables (cultural looseness, rule of law, national IQ, and support for military targeting of civilians), which we included for exploratory purposes.

^†^The Moral Machine website (moralmachine.mit.edu) was designed to collect data on the moral acceptability of decisions made by autonomous vehicles, and was deployed in June 2016. The website offers three interfaces other than the Classic mode, which are not discussed in this paper.
