# Supplementary material for: Universals and variations in moral decisions made in 42 countries by 70,000 participants
Source: Proc Natl Acad Sci U S A. 2020 Jan 21;117(5):2332–7. doi: 10.1073/pnas.1911517117 (PMC7007553; doi:10.1073/pnas.1911517117)
Supplement: Supplementary File [file pnas.1911517117.sapp.pdf]

Universals and variations in moral decisions made in 42 countries by 70,000 participants – Supplemental Information

Edmond Awad[a,b,1], Sohan Dsouza[b], Azim Shariff[c,1], Iyad Rahwan[b,d,e,1], and Jean-François Bonnefon[b,f,1]

[a] *Department of Economics, University of Exeter Business School, Exeter, EX4 4PU, UK*
[b] *The Media Lab, Massachusetts Institute of Technology, Cambridge, MA 02139, USA*
[c] *Department of Psychology, University of British Columbia Vancouver, BC V6T1Z4, Canada*
[d] *Centre for Humans & Machines, Max-Planck Institute for Human Development, Berlin 14195, Germany*
[e] *Institute for Data, Systems, and Society, Massachusetts Institute of Technology, Cambridge, MA 02139, USA.*
[f] *Toulouse School of Economics (TSM-Research), Centre National de la Recherche Scientifique, University of Toulouse Capitole, Toulouse 31015, France.*
[1] *To whom correspondence may be addressed. E-mail: e.awad@exeter.ac.uk, afshariff@gmail.com, rahwan@mpib-berlin.mpg.de, or jfbonnefon@gmail.com*

1 Demographics

After completing the 3-scenario session, users are presented with a survey of their demographic, political, and religious characteristics. This survey is optional. Out of $71.3K$ completed sessions, $45K$ surveys were taken. Surveys are taken from $20.6K$ distinct locations. Globally, the sample shows the expected biases: 75% male, 73% college-educated, 75% younger than 32. Overall, 36% of survey takers are young college-educated men. The sample is predominantly progressives (63 on a 100-scale from conservative to progressive) and secular (27 on a 100-scale from non religious to religious). Once we remove countries in which we have less than 200 surveys, we end up with data from 22 countries and $17.4K$ distinct locations.

Figure S1 shows the demographic, political, and religious characteristics of participants from these 22 countries. One can see that while generally skewed to one side, Gender, Age distributions, Politics distributions and Religiosity distributions are rather stable across countries. On the other hand, percentage of College degree holders in a country does not seem to reflect a representative sample of the population of a country. For example, the table shows disproportionate highly-educated samples from places like Mexico, Ukraine and China, suggesting that the data from these places might be distorted by self-selection.

www.pnas.org/cgi/doi/10.1073/pnas.1911517117

		Men (%)	College (%)	Age	Politics	Religiosity	N
Oceania	AU	73	69				413
Americas	BR	69	67				1,145
	CA	70	71				679
	US	71	74				3,414
	MX	76	87				242
Europe	CZ	81	52				208
	IT	80	56				367
	DE	78	60				1,938
	AT	70	61				222
	PL	77	66				424
	BE	75	67				291
	CH	77	67				279
	FR	72	74				2,512
	RU	78	78				1,397
	ES	83	79				646
	GB	74	80				1,110
	NL	77	84				340
	UA	75	85				455
Asia	HK	77	70				208
	TR	72	76				459
	TW	80	86				330
	CN	75	90				328

Figure S1: **Demographic, political, and religious characteristics of participants.** Gender, Age distributions, Politics distributions and Religiosity distributions are skewed, but stable across countries. Percentage of College degree holders differs across countries, and it may reflect a self-selected highly-educated sample in some countries.

Figure S2 shows the Cohen's h distance between sub-populations defined based on each demographic trait for each scenario in each country (the dotted line means no difference). Visual inspection suggests that demographics do not strongly impact responses, to the exception of the effect of a college education on Footbridge.

Table S1 reports the results of three multilevel logistic regressions (one for each scenario variant) predicting the proportion of participants endorsing sacrifice, based on gender (binary), age (standardized), education (college-educated or not), politics (standardized) and religiosity (standardized), allowing for a different intercept for each country.

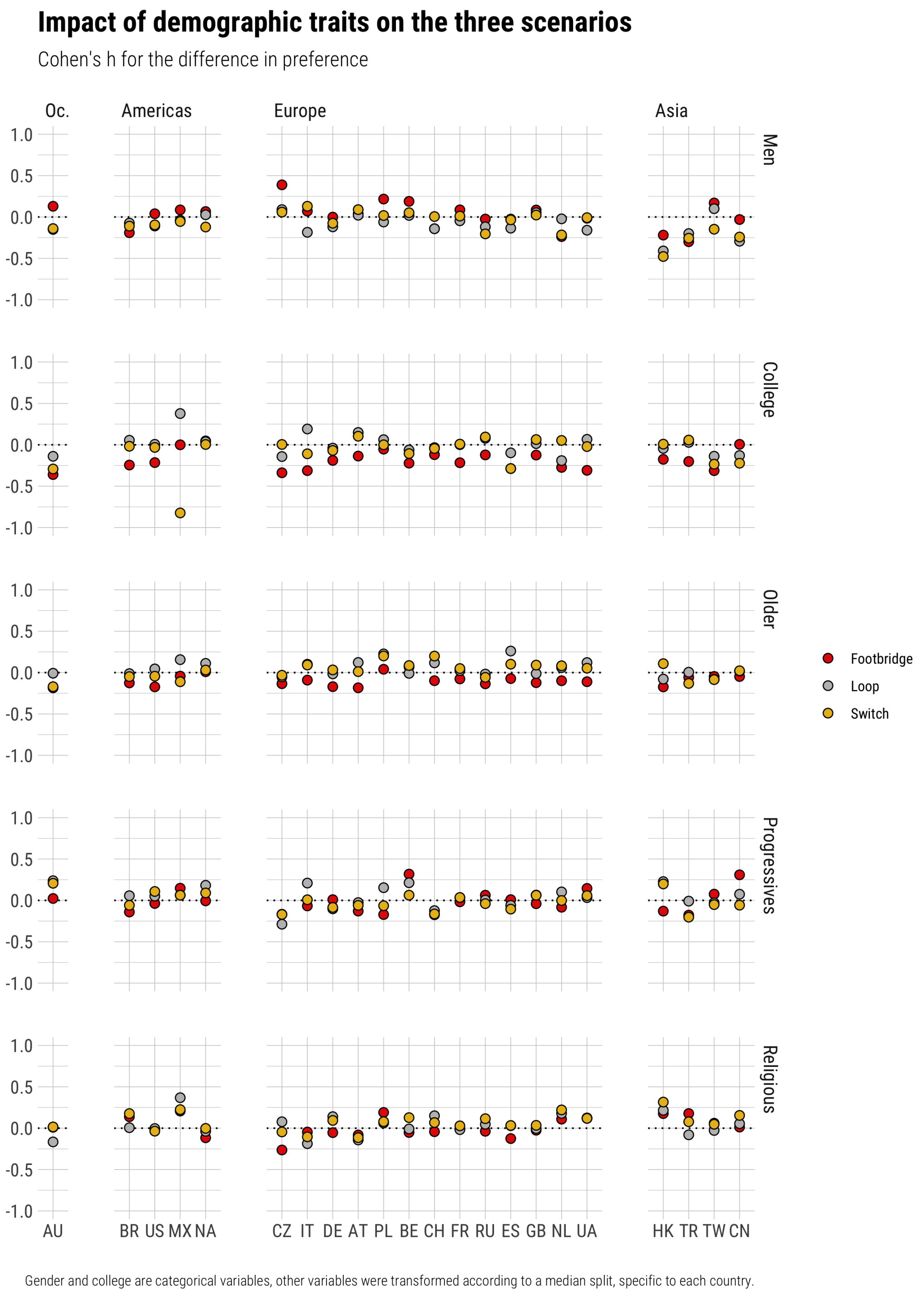

Figure S2: **Cohen's h distance between sub-populations defined based on each demographic trait for each scenario in each country.** The dotted line means no difference. Age and college education seem to robustly impact Footbridge. Sub-populations defined by the remaining characteristics seem to have no strong or systematic difference.

Table S1: Multilevel regressions allowing for different intercepts per country. The variables *Women* and *College education* are binary, while the rest are continuous.

	Switch	Loop	Footbridge
Women	0.217***	0.189***	−0.057
	(0.047)	(0.040)	(0.034)
Age	0.032	0.062**	−0.066***
	(0.021)	(0.018)	(0.015)
College education	−0.135*	−0.026	−0.358***
	(0.047)	(0.039)	(0.034)
Politics	0.073**	0.056*	0.010
	(0.021)	(0.018)	(0.016)
Religiosity	0.047	0.004	0.013
	(0.021)	(0.018)	(0.016)
Constant	1.621***	1.004***	0.108
	(0.067)	(0.056)	(0.045)
N	19,951	19,664	19,544

Note: *p<0.01; **p<0.001; ***p<0.0001

2 Robustness checks

This section shows the estimates calculated in Figure 2 for different subgroups of participants. That is the estimate of sacrificing one to spare five in each of the three scenarios for each country. Subgroups are chosen to show that the estimates are robust to different user interface (UI) manipulations and some of the choices made by users when taking the survey. Figure S3 shows estimates for two subgroups: those who visited the website and did the survey using a desktop device and those who did it using a mobile device.

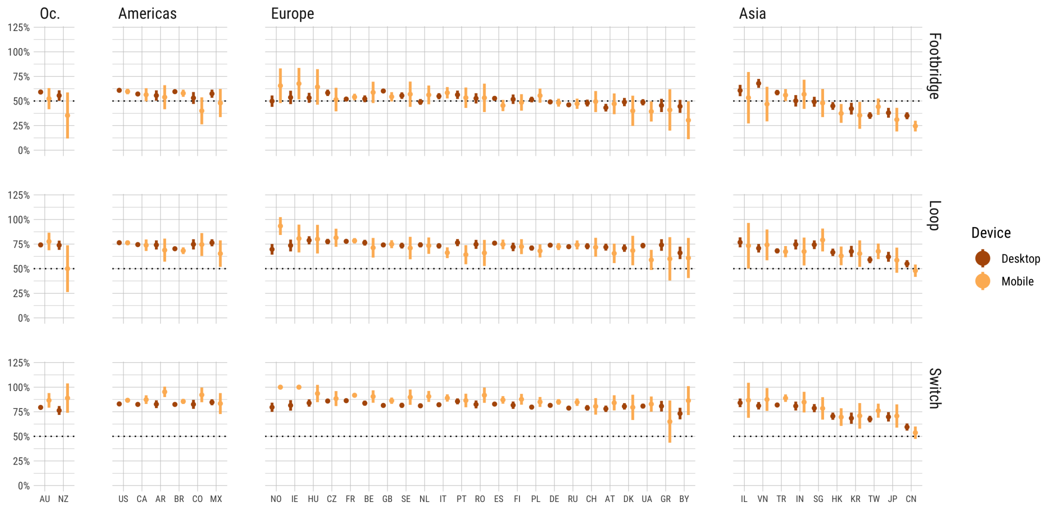

Figure S3: **Robustness Check: Device used (Desktop vs. Mobile).** Estimates remain stable regardless of whether respondents used desktop or mobile when completing the task.

Once a user visits the Moral Machine website, they can choose one of the ten available languages: Arabic, Chinese, English, French, German, Japanese, Korean, Portuguese, Russian, and Spanish. Figure S4 shows estimates for three subgroups: 1) Native speakers: someone from a country using the native language of that country (e.g., a user from US who is browsing in English, or a user from Japan who is browsing in Japanese). 2) Non-Native English: someone from a Non-English-speaking country using the English version (e.g., a user from Italy who is browsing in English). 3) Non-Native Other: someone from a country using a language that's neither English nor a native language of the country (e.g., a user from USA who is browsing in Spanish). This takes into consideration countries with multiple native languages (e.g., Canada with English and French as native languages).

Users who visit the website may choose to directly go to the survey. However, we know that before so doing, many had visited the "Judge" interface of the Moral Machine website, which is focused on the ethics of self-driving cars. One may wonder whether going through the moral dilemmas faced by self-driving cars have any carryover effect on the three scenarios. Fortunately, we can track the order in which users visited these two parts

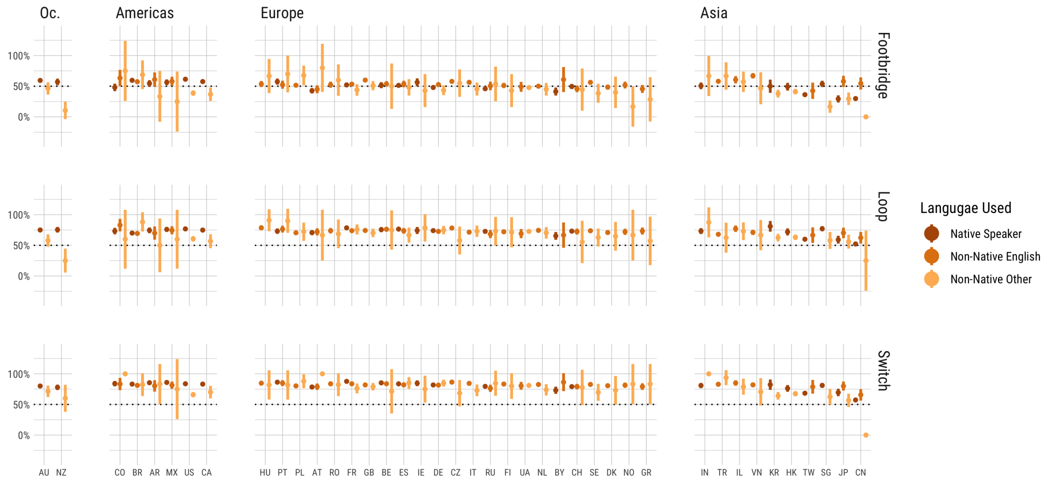

Figure S4: **Robustness Check: Language used (Native speaker vs. Non-Native English vs. Non-Native Other).** Estimates remain stable across three groups per country.

of the website. Figure S5 shows estimates for two subgroups: those who started the three scenarios before visiting the "Judge" interface (Before MM), and those who did it after visiting the "Judge" interface (After MM).

As explained in the main manuscript, each session is comprised of three scenarios: Switch, Footbridge, and Loop. The order of these scenarios is chosen randomly for each session. Previous work noted carryover effects between these three scenarios. Given this, it would be important to check for robustness of the results against the presentation order. Figure S6 shows estimates of each of the three presentation scenarios in each of the three possible orders: First, Second, and Third.

As explained in the main manuscript, each of the three scenarios has two decisions (corresponding to two outcomes): 1) Omission: do nothing, which would result in the trolley killing five people, and 2) Commission: take an action, which would result in sacrificing one person to spare five people. The presentation of these two outcomes is randomly chosen to be in one of two ways: 1) Sacrificing the one person is presented first (on the left side of the screen), followed by the other decision; leaving five to die (on the right side), and 2) vice versa. Figure S7 shows estimates of each of the three scenarios in each of the two possible presentation orders: sacrificing on the left, and sacrificing on the right.

Finally, in the previous section we showed demographic, political and religious characteristics of users, and whether they make any difference in terms of users' answers. Figure S8 shows that estimates of decisions made by those who took the survey correlate to those who did not take the survey, taken at the country level.

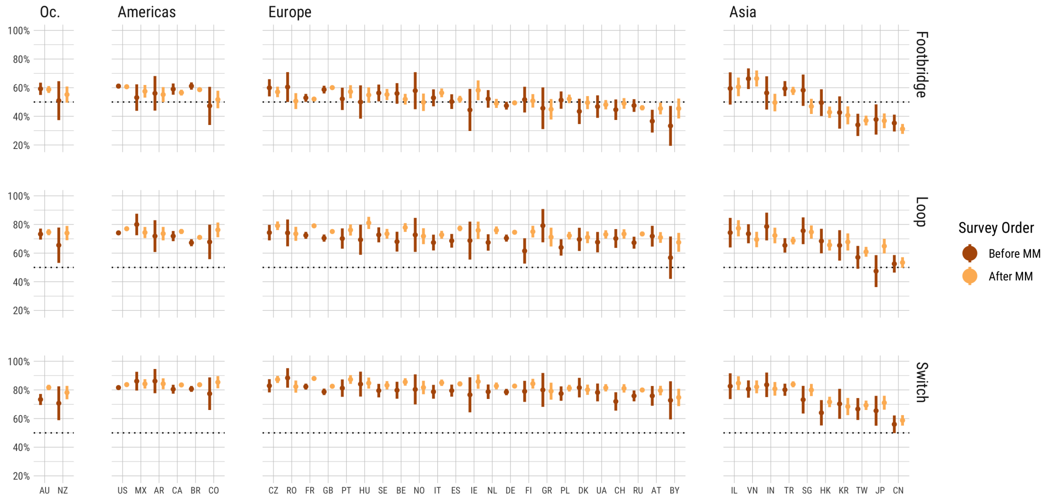

Figure S5: **Robustness Check: Carryover from Moral Machine (Before MM vs. After MM).** Estimates remain stable regardless of whether respondents had seen the Moral Machine's "Judge" interface before completing the task or not.

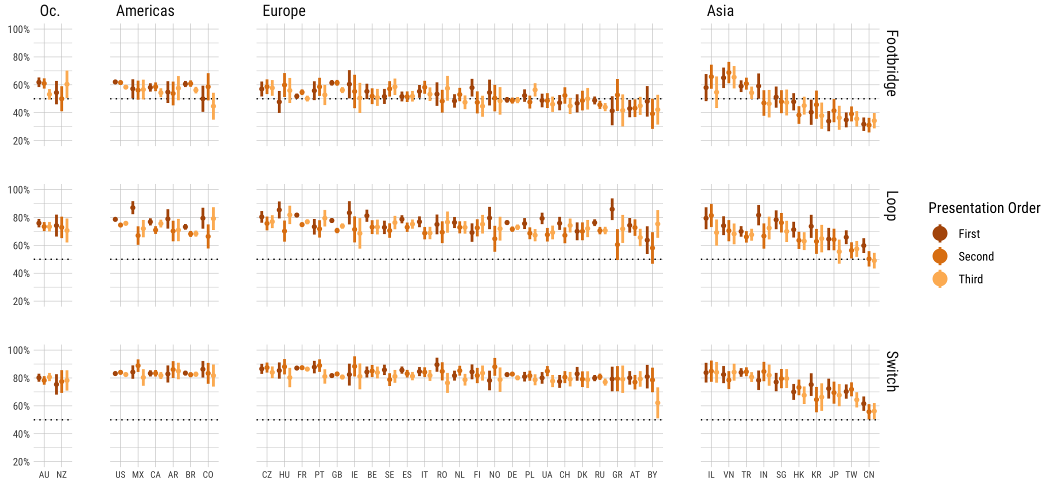

Figure S6: **Robustness Check: Scenario Order (First vs. Second vs. Third).** Estimates remain stable regardless of whether the scenario was presented first, second or third.

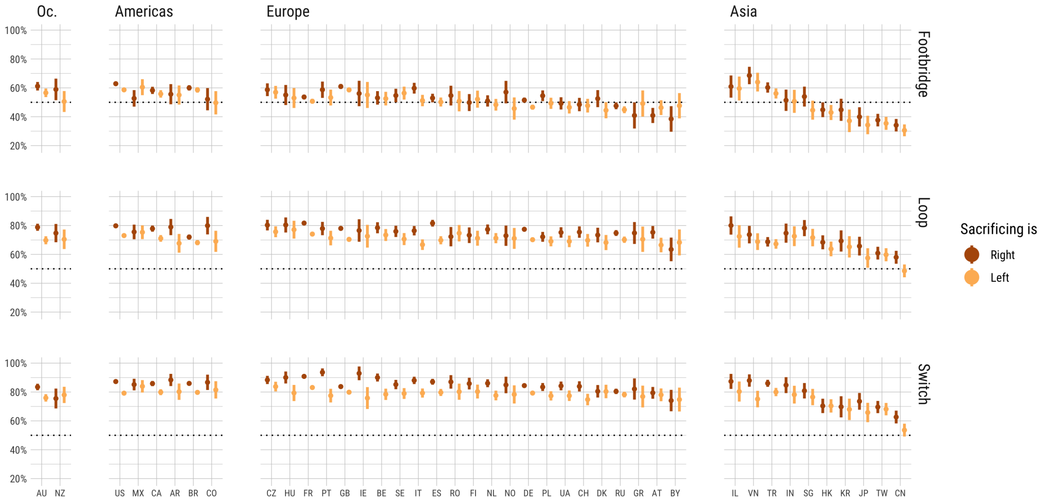

Figure S7: **Robustness Check: Outcome Order (Sacrificing one is on the Right vs. on the Left).** Estimates remain stable regardless of whether the decision to sacrifice one in order to spare five is on the right side or the left side of the screen.

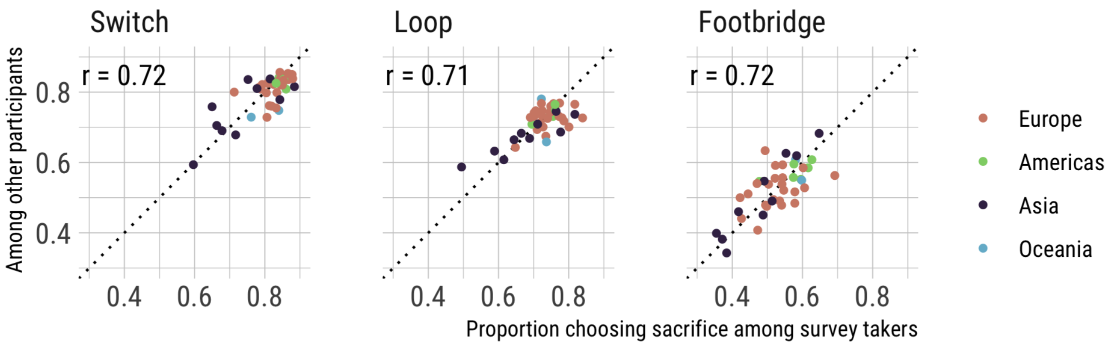

Figure S8: **Robustness Check: Survey Taking (Survey takers vs. Other participants).** Country-level estimates for users who took the (demographic, political and religious) survey at the end correlate to those for their counterparts who did not take the survey.

3 Country-level Correlations

3.1 Matrix

Figure S9 shows the matrix of zero-order correlation between our measures of interest (Switch, Loop, and Footbridge), the country-level variables analyzed in the main text (Relational Mobility, Individualism, GDP, and Religiosity), and the three additional country-level variables we considered for exploratory purposes (IQ, Rule of Law, and Cultural Looseness).

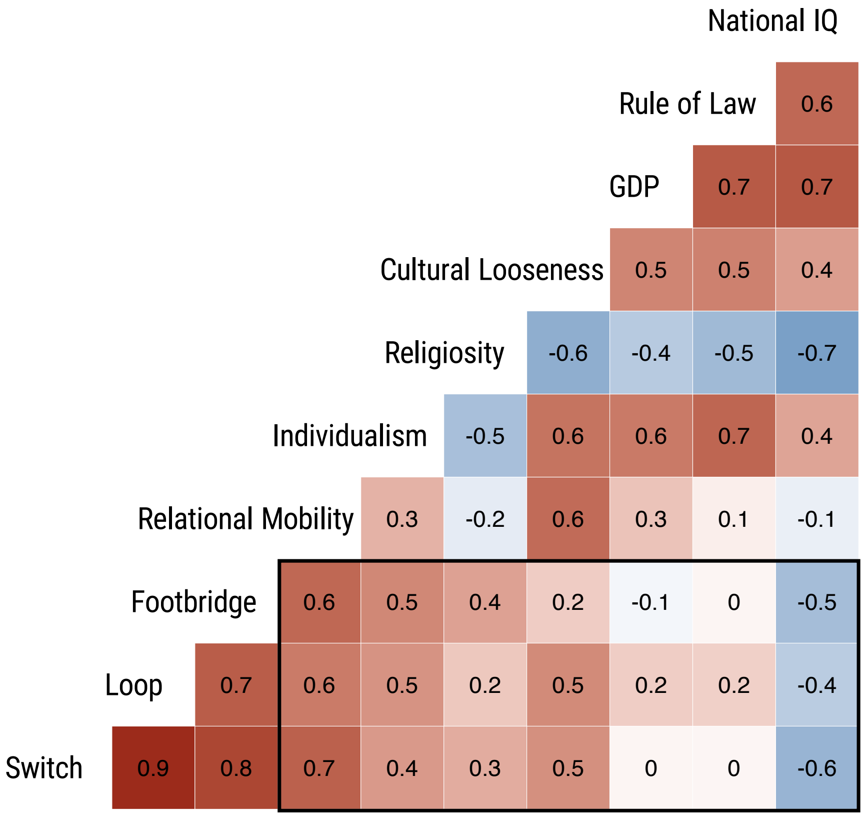

Figure S9: **Correlation matrix for measures of interest and country-level variables.**

3.2 Primary Indicators

- Log Gross Domestic Product (GDP) per capita. The GDP per capita data is purchasing power parity-adjusted GDP per capita in constant 2017 international dollars from the World Economic Outlook Database by International Monetary Fund (1).

- Relational mobility. Informed by papers by Thomson et al.(2) and Everett et al. ((3), we had made *a priori* predictions about the relationship between moral decision-making in the trolley dilemmas and relational mobility. This metric was calculated by Thomson et al. (2) and refers to the degree of fluidity versus stability in interpersonal relationships. A society with low relational mobility is characterized by stable long-term relationships (in social, work and romantic life) that are the more likely to be the product of circumstance, and more difficult to replace. Cultures with high relational mobility, in contrast, involve more freedom to choose and change relationships. Thomson et al. (2) produced national estimates of relational mobility using surveys administered to over 18,000 Facebook users, across 46 countries (and reported means for the 39 countries with >100 completed surveys).

- Individualism: Given previous cross-national results linking utilitarian moral decision-making to individualism in our earlier work (4), we elected to again test this relationship here. This indicator is based on Hofstede (5) (retrieved from `http://geert-hofstede.com/,accessed28.10.2015`). According to Hofstede, individualism (vs collectivism) captures the following underlying concept: "The high side of this dimension, called individualism, can be defined as a preference for a loosely-knit social framework in which individuals are expected to take care of only themselves and their immediate families. Its opposite, collectivism, represents a preference for a tightly-knit framework in society in which individuals can expect their relatives or members of a particular in-group to look after them in exchange for unquestioning loyalty. A society's position on this dimension is reflected in whether people's self-image is defined in terms of "I" or "we." The indicator is based on 30 questions and largely rests on IBM employees around the world. Many have remarked on the similarity between relational mobility and individualism. However, the two are very distinct constructs and only modestly correlate at the cross-national level (r=0.32). Relational mobility is a feature of the social ecology, and its measure asks about perceptions of people's behavior and options for forming and leaving relationships. Individualism, in contrast, is a feature of cultural norms and mindsets, with its measures asking about people's preferences and values. Thomson et al. (2) argue that the primary causal pathway is that features of the environment (e.g. threats and subsistence patterns) affect relational mobility, which in turn affects cultural norms like individualism.

- Religiosity: Previous research has found that religiousness and deontological decision-making are strongly related at the individual level (6; 7), we predicted that we might see pronounced country-level effects. These data were compiled from country means for the percentage of people who choose "Very important" to the item asking "For each

of the following aspects [Religion], indicate how important it is in your life. Would you say it is:" in the World Values Survey (8). However, just as we saw minimal individual-level relationships between subject religiosity and ethical decision-making (Table S1, Figure S2), the country-level relationships were also modest (Figure S9). Where they did appear, they ran opposite to predictions based on individual-level research, with more religious countries reporting more utilitarian preferences.

3.3 Secondary Indicators

In addition to the four variables above, which are discussed in the main text and for which we had made a priori predictions, we also tested three other theoretically-relevant variables in a more exploratory sense:

- Rule of Law: Rule of Law had shown a significant relationship with a preference to spare more characters in our earlier research on cross-national differences in moral decision-making (4), and we thus again investigated the relationship here. The Rule of Law index was compiled by the World Bank Group as part of their World Governance Indicators. It amalgamates dozens of metrics measuring features such as the confidence in the police, fairness and speed of the judicial, frequency of crime, etc.

- IQ: Previous research has tied deontological vs utilitarian ethical decision-making to cognitive abilities. For example, Moore, Clark and Kane (9) showed the individual differences in working memory capacity predicted utilitarian choices in moral scenarios. Since there are few broadly available cross-national measures of cognitive ability, we used IQ as a loose proxy. The measure was compiled by Lynn and Meisenberg (10) and validated against measures of education attainment. While Footbridge (r=-0.51), Loop (r=–0.39), and Trolley (r=-0.58) all showed strong correlations with national-level IQ, each of these relationships were driven by the difference between East Asian countries (which tend to rank highly on national IQ, and score low on utilitarian decision-making) and the countries in the rest of the world.

- Cultural Looseness/Tightness: The concept of Cultural Looseness and Tightness, which refers to how much cultural latitude there is for variability in and deviation from norms and values, shows considerable cross-national overlap with both individualism (r=0.60) and relational mobility (r=0.63)–though, again, Thomson et al. (2) argue that, as a feature of the social ecology, relational mobility causally precedes Cultural Looseness/Tightness. Culturally loose societies tend to tolerate greater heterogeneity, whereas tight cultures enforce conformity in attitudes and norms and thus groups end up more homogeneous in the positions they hold, or at least express. Uz (11) calculated the Looseness/Tightness scores for 68 countries, based on the degree of variation within countries on agreement on a set of values and practices. There are moderate relationships between Cultural Looseness/Tightness and responses on the ethical dilemmas, though, as with IQ, these are primarily due to differences between East Asian (relatively tight cultures with relatively low rates of utilitarian decision-making) and other countries.

3.4 Tertiary Indicators

In response to a reviewer comment, we conducted one more analysis to investigate the ecological validity of the trolley decisions.

- Support for the Military Targeting of Civilians: In order to explore whether utilitarian decision-making in the trolley dilemmas predicted support for other utilitarian attitudes at the national level, we tested whether there was a relationship between the trolley dilemmas and the percentage of the population that chose "Sometimes Justified" for the statement: "For the military to target and kill civilians is sometimes justified, while others think that kind of violence is never justified" (12). The relationship with the Footbridge dilemma, in which sacrificing the uninvolved large man is most closely analogous to the sacrificing of uninvolved civilians, was particularly strong (r=0.50; Fig S10), whereas those for Loop (r=0.37) and Trolley (r=0.22) were more modest. These relationships persist when controlling for GDP per capita. Moreover, unlike the relationships between IQ and the dilemmas, these relationships were not solely driven by the differences between East Asian and other countries, as the relationships appeared among both clusters. These connections with the support for the sacrifice of civilians to serve utilitarian ends in military endeavours provide some of the best evidence for the group-level ecological validity of trolley problems–which has been a matter of recent debate (13; 14).

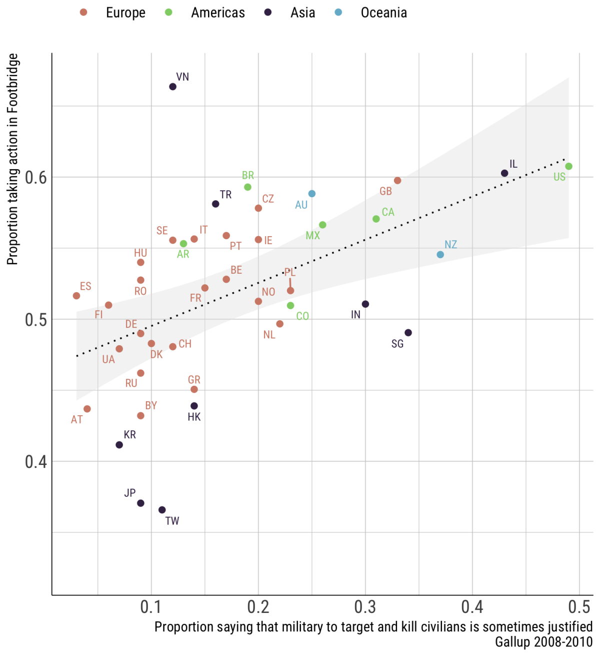

Figure S10: **Association between country-level utilitarian decisions in the footbridge dilemma and percentage of the population that reports that the military targeting of civilians is sometimes justified**

References

[1] International Monetary Fund. World economic outlook database. http://www.imf.org/external/pubs/ft/weo/2017/01/weodata/index.aspx (2017).

[2] Thomson, R. *et al.* Relational mobility predicts social behaviors in 39 countries and is tied to historical farming and threat. *Proceedings of the National Academy of Sciences* **115**, 7521–7526 (2018).

[3] Everett, J. A., Pizarro, D. A. & Crockett, M. Inference of trustworthiness from intuitive moral judgments. *Journal of Experimental Psychology: General* **145**, 772 (2016).

[4] Awad, E. *et al.* The moral machine experiment. *Nature* **563**, 59–64 (2018).

[5] Hofstede, G. *Culture's consequences: Comparing values, behaviors, institutions and organizations across nations* (Sage publications, 2003).

[6] Piazza, J. & Landy, J. " lean not on your own understanding": belief that morality is founded on divine authority and non-utilitarian moral thinking. *Judgment and Decision making* **8**, 639–661 (2013).

[7] Piazza, J. & Sousa, P. Religiosity, political orientation, and consequentialist moral thinking. *Social Psychological and Personality Science* **5**, 334–342 (2014).

[8] Inglehart, R. *et al.* World values survey: All rounds - country-pooled datafile 1981–2014. *Madrid: JD Systems Institute.* **1**, 2018 (2014). URL http://www.worldvaluessurvey.org/WVSDocumentationWVL.jsp.

[9] Moore, A. B., Clark, B. A. & Kane, M. J. Who shalt not kill? individual differences in working memory capacity, executive control, and moral judgment. *Psychological science* **19**, 549–557 (2008).

[10] Lynn, R. & Meisenberg, G. National iqs calculated and validated for 108 nations. *Intelligence* **38**, 353–360 (2010).

[11] Uz, I. The index of cultural tightness and looseness among 68 countries. *Journal of Cross-Cultural Psychology* **46**, 319–335 (2015).

[12] Gallup. Views of violence: What drives public acceptance and rejection of attacks on civilians 10 years after 9/11. Tech. Rep. (2011).

[13] Bostyn, D. H., Sevenhant, S. & Roets, A. Of mice, men, and trolleys: Hypothetical judgment versus real-life behavior in trolley-style moral dilemmas. *Psychological science* **29**, 1084–1093 (2018).

[14] Plunkett, D. & Greene, J. D. Overlooked evidence and a misunderstanding of what trolley dilemmas do best: Commentary on bostyn, sevenhant, and roets (2018). *Psychological science* 0956797619827914 (2019).
